# High-Performance Magnetic-core Coils for Targeted Rodent Brain Stimulations

**DOI:** 10.34133/2022/9854846

**Published:** 2022-03-05

**Authors:** Hedyeh Bagherzadeh, Qinglei Meng, Hanbing Lu, Elliott Hong, Yihong Yang, Fow-Sen Choa

**Affiliations:** ^1^Department of Computer Science and Electrical Engineering, University of Maryland, Baltimore County, Baltimore, MD 21250, USA; ^2^Neuroimaging Research Branch, National Institute on Drug Abuse, National Institutes of Health, Baltimore, MD 21224, USA; ^3^Maryland Psychiatric Research Center, Department of Psychiatry, University of Maryland School of Medicine, Baltimore, MD 21201, USA

## Abstract

*Objective and Impact Statement*. There is a need to develop rodent coils capable of targeted brain stimulation for treating neuropsychiatric disorders and understanding brain mechanisms. We describe a novel rodent coil design to improve the focality for targeted stimulations in small rodent brains. *Introduction*. Transcranial magnetic stimulation (TMS) is becoming increasingly important for treating neuropsychiatric disorders and understanding brain mechanisms. Preclinical studies permit invasive manipulations and are essential for the mechanistic understanding of TMS effects and explorations of therapeutic outcomes in disease models. However, existing TMS tools lack focality for targeted stimulations. Notably, there has been limited fundamental research on developing coils capable of focal stimulation at deep brain regions on small animals like rodents. *Methods*. In this study, ferromagnetic cores are added to a novel angle-tuned coil design to enhance the coil performance regarding penetration depth and focality. Numerical simulations and experimental electric field measurements were conducted to optimize the coil design. *Results*. The proposed coil system demonstrated a significantly smaller stimulation spot size and enhanced electric field decay rate in comparison to existing coils. Adding the ferromagnetic core reduces the energy requirements up to 60% for rodent brain stimulation. The simulated results are validated with experimental measurements and demonstration of suprathreshold rodent limb excitation through targeted motor cortex activation. *Conclusion*. The newly developed coils are suitable tools for focal stimulations of the rodent brain due to their smaller stimulation spot size and improved electric field decay rate.

## 1. Introduction

Transcranial magnetic stimulation (TMS) is an FDA-approved treatment for major depression disorder (MDD), migraine, and obsessive-compulsive disorder (OCD) [1–4]. Since it is noninvasive and rarely induces adverse effects, TMS has also been used as a research tool [5]. Working together with imaging modalities, such as electroencephalogram (EEG) and functional magnetic resonance imaging (fMRI), it has been used for mapping stimulation-elicited brain activity [6, 7]. TMS has been extensively used to investigate therapeutic efficacies of various neurological and neuropsychiatric disorders [8–10] and to assess neurobiological mechanisms underlying brain functions [11–13].

One of the well-known limitations of using TMS coils is the issue of depth-focality tradeoff. Undesirable stimulation of nontargeted regions, especially for deeper regions in the brain, can affect the TMS clinical outcome and possibly increase the risk of seizures or other adverse side effects [14–17]. The negative impacts of this tradeoff are more distinct in animal studies due to the relatively smaller brain sizes. To precisely activate a brain subregion in a smaller rodent, coil emission patterns with reduced spot size are required. Several previous works have been focused on introducing new coils with improved depth-focality performance and reduced spot size. These were done through analytical models or numerical simulations using either the finite element method (FEM) or finite difference method (FDM) [15, 18–22].

Preclinical animal experiment is an essential step of translational TMS research. The advantages of animal studies include the homogeneity across individuals, availability of various disease models, and translational relevance to human TMS in safety, synaptic plasticity, neuronal connectivity, and cortical organization [23–25]. Currently, there is no commercial TMS system capable of focusing the stimulation volume down to the functionally distinguishable rodent brain regions.

Commercial TMS coils are generally air-core-based and operate at kiloampere levels to induce suprathreshold stimulation. The theoretical analysis suggested that when the coil diameter is reduced 5 times, the electric field strength drops by a factor of 25, resulting in a significant loss in the electric field’s radial propagation [26]. Thus, scaling down a TMS coil from human to rodent would require an exceedingly high current. The power requirement, Ohmic heating, and electromagnetic stress pose daunting engineering challenges. The efficiency limitation practically restricts an air-core-based coil diameter to no smaller than 2.5 cm [14]. As a result, the smallest commercial coils usually have a coil diameter of around 2.5 cm. Since reducing the coil size beyond that is challenging, a significant reduction to the stimulation focal spot size below the millimeter range is highly unlikely. Thus, there is a strong demand for developing new TMS coil designs capable of stimulating a focal brain region, specifically for smaller animals like rodents.

Several studies reported the designs of rodent-specific TMS coils [23, 27, 28]. In general, these coils either stimulate a large portion of the rodent brain or the induced field is too weak to reach suprathreshold brain stimulation, as Bagherzadeh and Choa [21] pointed out. High permeability materials can enhance magnetic field strength and have been applied to design TMS coils for humans [29, 30]. While using ferromagnetic cores can enhance the magnetic field strength, it also increases the inductance of the coil, and with the same driver voltage, the coil current and the delivered power will reduce. On the other hand, using the ferromagnetic core, not only can increase the magnetic field but also produce a geometric redistribution of the magnetic field and make the field more focused and easier to reach the activation threshold [31–33]. One potential issue with using ferromagnetic cores is the magnetic saturation at high currents, which can lead to undesirable loading of the coil driving circuit. The key to avoid this saturation is using ferromagnetic material with a high saturation threshold [16, 29, 34]. Our research was based on these concepts to design a TMS coil for rodents and achieved suprathreshold focal stimulation of the mouse brain [35]. Nevertheless, several important questions are still unanswered. Since the magnetic flux diverges rapidly outside the magnetic core, the study by Epstein and Davey [29] suggested that the effects of magnetic core materials were constrained to the brain regions close to the coil. Considering the small penetration depth required for rodents’ brain stimulation, it is unknown how the magnetic core behaves in terms of penetration depth-field focality tradeoff, a critical metric in assessing TMS coils’ performance. Furthermore, we developed an innovative wire-wrapping method to break the circular symmetry of the field distribution pattern, inducing an elliptical field shape with a simple and single-element structure.

Our proposed TMS coil design provides a unique research tool for TMS studies due to their enhanced depth-spread performance. We can overcome the low-efficiency problem encountered by the small diameter coils using a ferromagnetic core. A ferromagnetic core prevents the magnetic flux leakage out of the coil wall and enhances the field strength. We also generated a tightly focused electric field distribution in a homogenous spherical head model with the proposed coil design. The design demonstrated smaller stimulation spot size, improved electric field decay rate, and up to 60% lower energy requirement for rodent brain stimulation. The results were validated with the finite element method (FEM) simulations and experiments.

## 2. Results

### 2.1. Coil Prototype Experimental Results

To study the performance of the proposed coils and compare them with existing and commercial coils, prototypes of the angle-tuned coils were fabricated by wire-wrapping over a 3D-printed holder. The characterization of the coil performance was done by measuring the induced electric field strength at different depths using a vector-field probe. The commercial coil used for this comparison was the 70 mm figure-8 Magstim coil. The details for the experimental setup are provided in the “Materials and Methods” section.

**Figure 1:**
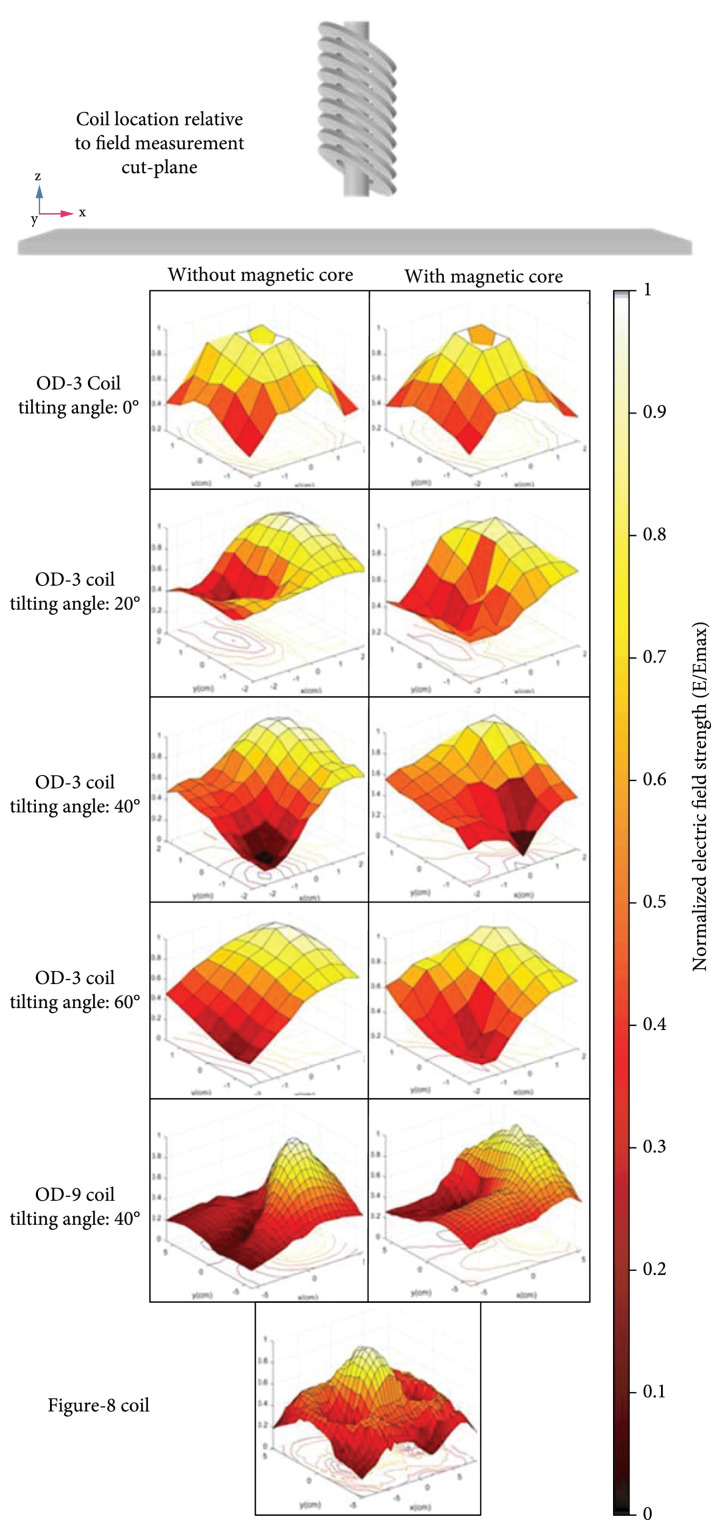
Electric field distribution through measurements for the proposed coils with different tilting angles with and without the magnetic core based on the experimental measurements performed in the air medium. The proposed coil and the magnetic core were placed perpendicular to the field measurement (head) cut-plane. All the data is normalized to the highest electric field intensity to compare the spatial distribution of two different angle-tuned coil sizes (OD-3 and OD-9) with different tilting angles with a commercial figure-8 coil. The grid size for the OD-3 coils is 4 cm×4 cm, while for OD-9 and Figure-8 coil, it is 10 cm×10 cm.

**Figure 2:**
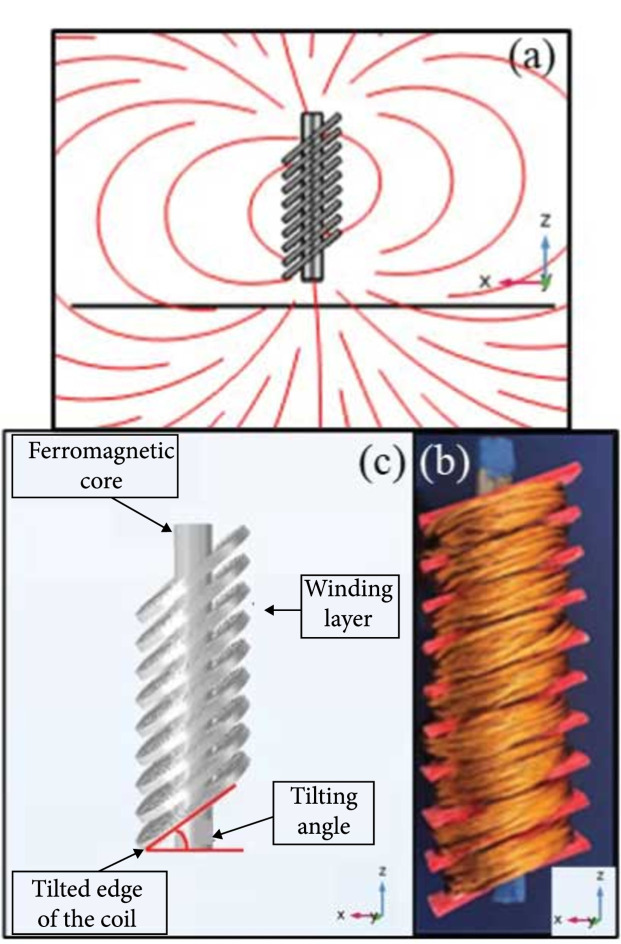
The proposed coil design with a magnetic core. (a) The coil placement schematic for experiments and simulations. The coil and core axis are perpendicular to the measurement cut-plane (head plane). The red lines demonstrate the streamline of the generated magnetic field. (b) Experimentally fabricated coil based on a wire-wrapped 3D printed coil holder. The alignment of the coil is shown with the coordination axis. (c) The FEM simulation model.

Figure 1 shows the measured electric field distributions at a 1.5 cm distance from the coil tips with and without the magnetic core obtained from the experimental setup. The proposed coil and the magnetic core were placed perpendicular to the field measurement (head) cut-plane, as shown in Figure 1. All the measurements were performed in the air for comparison purposes and to analyze the spatial distribution of the induced electric field. We used a novel electric field probe with high sensitivity and directivity designed in our lab [36]. The OD-9 and figure-8 coils have a larger size, and their emission field plots are composed of more measurement points, so the measured field distribution has a denser grid. From these normalized measurement results, we can conclude that for the angled ring coils, the peak of the field distribution is very close to the tilted edge of the coil (defined as the lowest point of the coil body along the z-direction as demonstrated in Figure 2(c)) and the hot spot of the figure-8 coil is located at the center of the coil. Increasing the tilting angle results in a sharper peak at the coil’s edge, which means that the spread of the induced electric field is reduced. The presence of the magnetic core can sharpen the observed field distribution peak and further reduce the spread of the electric field. This is because the magnetic core guides the magnetic flux with a more substantial density along the core axis direction (central axis of the coil). At the same time, the tilting angle pushes the field toward the tilted edge of the coil. This phenomenon results in a slight shift of the electric field’s peak location toward the core’s alignment, i.e., the coil’s central axis, compared with the case that no magnetic core is used.

**Figure 3:**
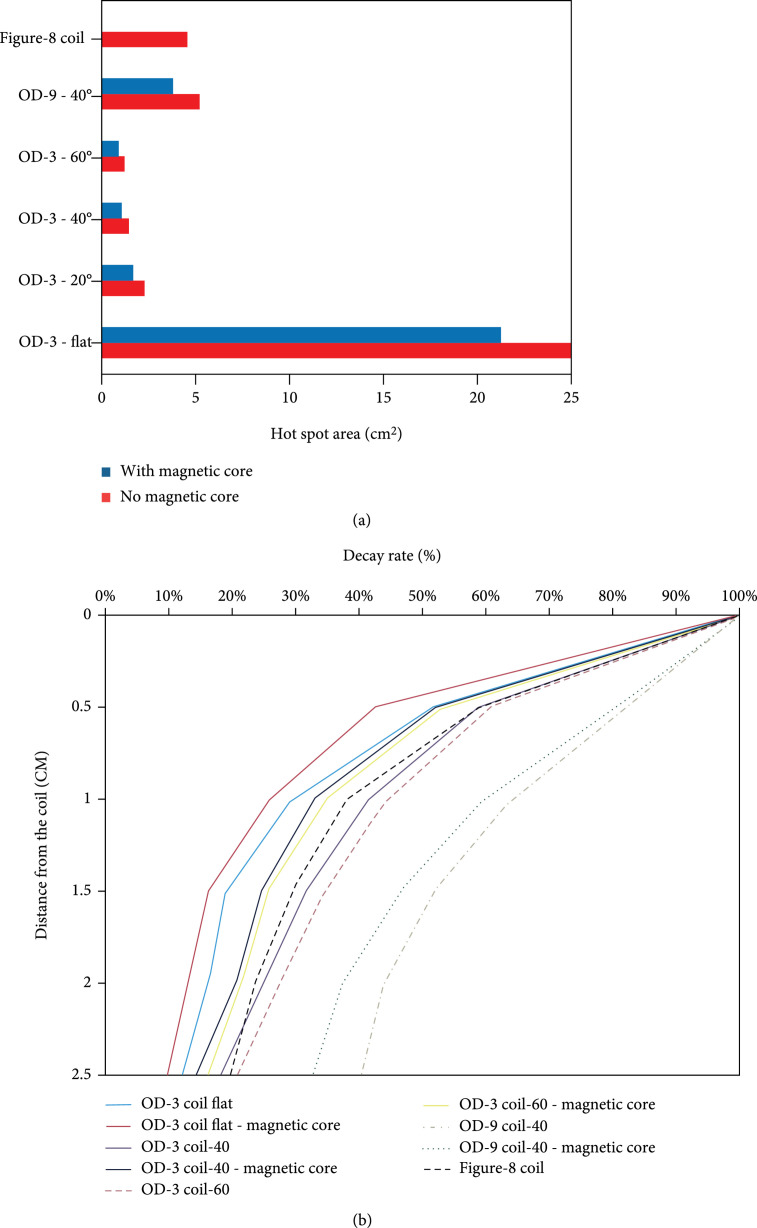
(a) Hot spot size of the proposed coils with and without the magnetic core at the distance of 1.5 cm from the surface of the coil measured using the fabricated prototypes; (b) electric field strength decay rate (in percentage) as a function of depth for the proposed coils with and without the magnetic core demonstrated as a percentage of the maximum electric field encountered near the coil. The data was obtained through the experimental setup.

Figure 3 shows the performance of the proposed coils evaluated by the hot spot area and the electric field decay rate defined here as the percentage of the electric field intensity at different depths to the electric field intensity at the bottom surface of the coil. Tilting the coil can significantly reduce the hot spot area, while adding the magnetic core further improves the coil’s focality. For all the coils, the magnetic core reduces the hot spot area by about 20%. The hot spot area, in this section, is defined as the area that has experienced 90% or more of the maximum electric field at 1.5 cm away from the coil’s surface. The addition of the magnetic core and tilting the coil to 60 degrees resulted in a 95% reduction of the hot spot size compared to the flat circular coil without a magnetic core.

At different depths, the coils’ electric field intensity was measured and normalized to the peak electric field measured near the coil surface. The normalized value is presented as the electric field decay rate for different coils at different depths, as shown in Figure 3(b). It shows that coils with higher tilting angles have slower decay rates. When comparing the decay rates of OD-3 and OD-9 coils, we can also observe that coils with a larger aperture have a smaller field divergence and a slower electric field decay rate. Adding the ferromagnetic core to the proposed coils increases the decay rate by about 10%, which means that these cores can reduce the spread but restrict the penetration depth. Nevertheless, with and without the ferromagnetic core, the tilted coils demonstrate a superior decay rate than the commercial 70 mm figure-8 coil. By combining the effects of using magnetic cores and angle-tuned coils, the proposed TMS coils can achieve a minimal stimulation spot size that is very promising for rodent brain stimulation.

### 2.2. FEM Simulation Results

#### 2.2.1. Depth-Spread Analysis

We have demonstrated the enhanced spatial distribution and performance of angle-tuned coils with and without the ferromagnetic core in air through experimental methods. To analyze the depth-spread performance of the proposed coils and compare them with existing coils, we referred to the depth-spread tradeoff profile plot introduced by Deng et al. [14]. The proposed model is a homogenous sphere that provides a steady platform to compare the depth-focality performance of the coils. Since the electric field intensity is normalized to the maximum electric field, the spatial characteristics of the electric field solely depend on the geometry and location of the coil [14, 37, 38]. Also, the sphere’s homogeneity results from the radial changes in conductivity not affecting the electromagnetic induction in a sphere [39]. The penetration depth and the spread in these models are counted from 1.5 cm away from the head’s surface, right at the “cerebral surface.” In all the models, the coil is located 0.5 cm above the head’s surface, representing the effect of about 0.5 cm thick insulating protection coating of TMS coils. Each coil’s absolute electric field strength values can be modulated through the TMS stimulator output and hold no significance in the spatial distribution.

**Figure 4:**
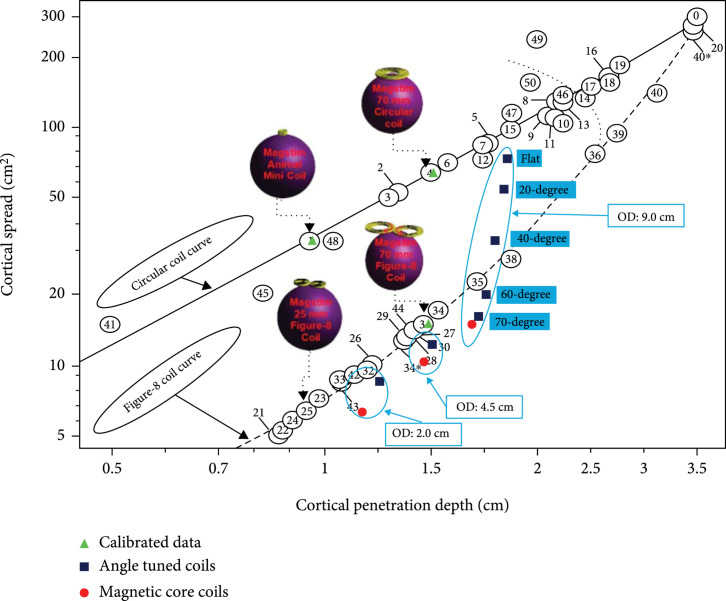
Effects of tilting angle and magnetic core on the depth-spread performance of the circular coils. The cortical spread and penetration depth considered here is calculated, starting from 1.5 cm from the head’s surface. O.D. represents the outer diameter of the tilted coils. All the simulations are based on spherical models and are presented for comparison purposes.

Figure 4 illustrates the performance of the proposed coils with and without the magnetic core. It should be noted that three of the coils used in Deng et al. [14] were simulated with COMSOL to calibrate and validate the results of the simulations. These 3 coils included coil #1 or the animal mini coil [40], coil #4 or the Magstim 70 mm circular coil [41], and coil #31 or Magstim 70 mm figure-8 coil [41]. These coils were selected randomly and solely for comparison and validation purposes. The angle-tuned coils have different outer diameters (9 cm, 4.5 cm, and 2 cm) and a winding thickness of 1 cm. We simulated different tilting angles for the 9 cm outer diameter (O.D.) coils, ranging from 0 to 70 degrees. For the other two coils (with 4.5 cm and 3 cm O.D.s), we only simulated the case of a 70-degree tilting angle. All coils have 5 layers of wire stacking.

As shown in Figure 4, increasing the tilting angle of the coils from 0 degrees (flat circular coil stack) to 70 degrees results in a significant reduction of field spread. When the tilting angle is over 40 degrees, the proposed coils’ depth-spread plot starts to pass below the figure-8 coil curve and accomplish better performance. Adding the magnetic core to the angle-tuned coils results in a further reduction of spread for all coils. However, a minor reduction (less than 5%) of penetration depth also occurs due to the insertion of an iron core. As shown in the figure, the ferromagnetic cores’ spread reduction effect is more significant for smaller coils. For the 9 cm O.D. coils, the iron core produced a spread reduction of around 6%, while for the 2 cm O.D. coil, the spread reduction is around 27%, making them suitable for targeted brain stimulation.

To show the enhancements in the performance of the proposed coils, we compared their depth-spread metrics (with and without the ferromagnetic core) with three commercial coils [14]: coil#1 or the animal mini coil [40], coil #31 or Magstim 70 mm figure-8 coil [41], and coil #25 or Magstim 25 mm figure-8 [42]. The angle-tuned coils with a 70-degree tilting angle are selected for this comparison since they demonstrate the smallest spread in respect to other tilting angles. Angle-tuned coil with an outer diameter of 2 cm (with and without the ferromagnetic core) demonstrates a 70% to 80% decrease in cortical spread with a 20% to 30% increase in cortical penetration compared to coil #1. Additionally, the same coil with ferromagnetic core has the same spread as coil #25 with increased cortical penetration of more than 25%. The significant improvements in the small angle-tuned coil’s depth-spread performance compared to existing animal coils promise a new tool for targeted brain stimulation. Furthermore, the depth-spread performance of angle-tuned coils with an outer diameter of 9 cm and 4.5 cm can be compared with coil #31, which is among the most conventional human TMS coils. Compared with coil #31, an angle-tuned coil with an outer diameter of 9 cm demonstrates the same spread with a 20% increase in cortical penetration, while angle-tuned coils have the same penetration depth with a 10% smaller spread. These analyses show the enhanced focality of the proposed coils for brain stimulation compared to the existing conventional TMS apparatus.

The smaller contact area, the increased focality, and the enhanced electric field spatial distribution of these coils make them suitable options to be considered for rodent studies. Specifically, the angle-tuned coil with an outer diameter of 2 cm and a ferromagnetic core demonstrated enhanced depth-spread performance compared to the commercial animal coils, as shown in Figure 4. The suitability of this coil for rodent brain stimulation is further analyzed in the following section.

#### 2.2.2. Required Energy and Hot Spot Analysis

**Figure 5:**
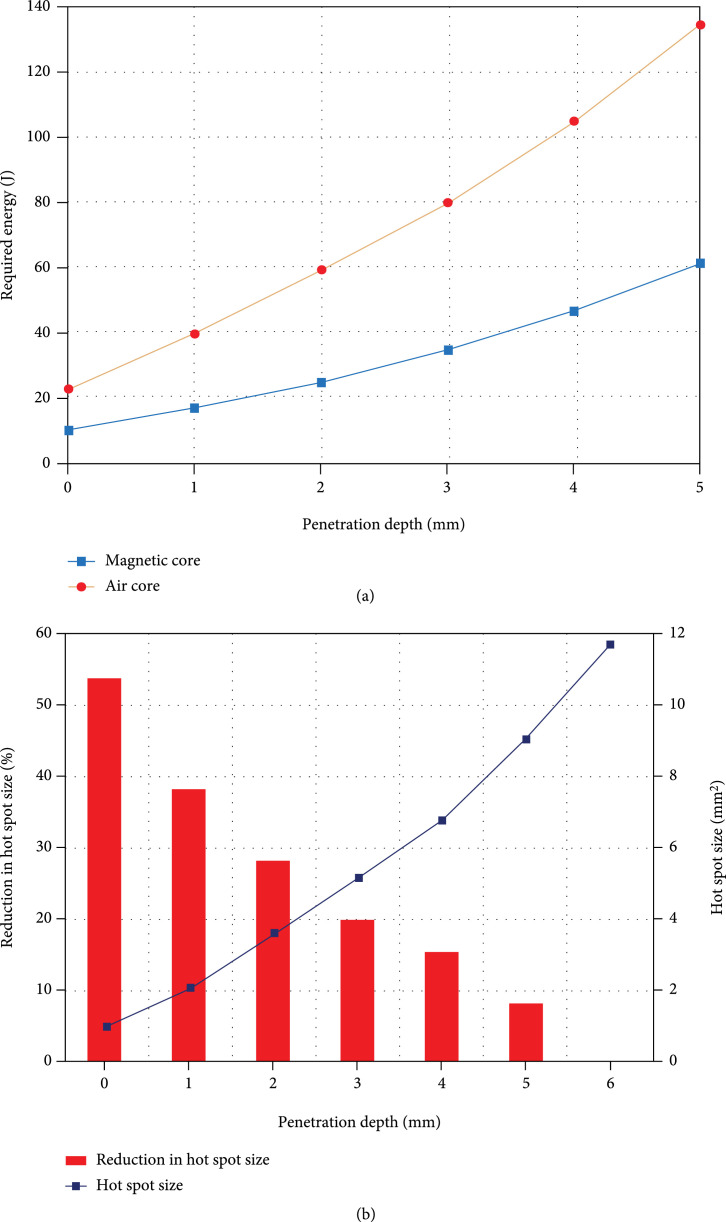
Effect of the magnetic core on the performance of angle-tuned coils. The coil considered for this analysis is a 70-degree tilted coil with an outer diameter of 2 cm. (a) Effect on the energy required for inducing 100 V/m at desired depths. The required energy in this plot is considered to induce 100 V/m in the desired depth from the head surface, considering a 2 mm thick insulation layer between the head model and the coil. (b) Effect of the ferromagnetic core on the stimulated hot spot area for the proposed coil at different depths in the brain. The hot spot area of the induced electric field in the brain is an area above the threshold with higher than 99% of the maximum electric field. The penetration depth is considered from the head surface with a 2 mm thick insulation between the coil and the head surface. The plot demonstrates the hot spot area of the coil with a ferromagnetic core and the reduction in the hot spot area by adding the ferromagnetic core to the coil structure.

Magnetic stimulation consumes a lot of energy in the process of high current and high voltage operations. Previous studies have also demonstrated that reducing the coil size will significantly increase energy consumption. It means that rodent coils require much higher energy, and as they become smaller, the energy requirement follows an inverse square-law and goes higher [43]. The energy efficiency analysis was done by considering the energy consumption required for a 2 cm O.D. and 70-degree tilted coil to reach a field threshold of 100 V/m [43] at different depths. In this analysis, a 2 mm thick high-voltage-insulation coating between the coil and the head surface is considered. Since adding ferromagnetic cores to existing coils can reduce their energy requirements [30], we have analyzed the coil’s performance with and without the introduced core. The coil’s energy consumption to reach the threshold at different depths is shown in Figure 5(a). The depths shown are counted from the head surface toward the brain’s inner parts. With a ferromagnetic core, the field strength can reach the threshold at low currents, reducing energy consumption by about 60% for all different depths up to 5 mm. For example, the energy required to induce 100 V/m at a depth of 2 mm, which is considered the cortical distance for mice [44], with the ferromagnetic core is 24.4 J compared to 59.3 J for the same coil without a core.

As shown in Figure 4, adding the magnetic core to the coil structure reduces the spread of the induced electric field. To show the focality improvement of the proposed coils, we simulated the hot spot size of a 2 cm O.D., 70-degree tilted coil with and without iron core at different depths. For rodent brain stimulation applications, 5 mm from the head surface into the brain is considered deep brain stimulation. A 2 mm thick insulation spacing between the coil and the head model was used in these calculations [27]. To evaluate the possibility of accomplishing a very small hot spot size for rodent brain stimulation [44], we consider that the only area above the threshold is the area with higher than 99% of the maximum electric field. Figure 5(b) shows the improvement (reduction) of the above-defined hot spot sizes at different depths by adding the magnetic core to the coil structure. We found that adding the magnetic core contains the induced electric field in the shallower depths. The hot spot size is reduced more significantly at depths closer to the coil since the magnetic flux diverges from the coil rapidly. For example, at the surface of the head, the presence of the magnetic core causes a more than 50% reduction in the spot size, while at 5 mm into the brain, this reduction drops to 8%. As the penetration depth increases to deeper than 6 mm, the coil with an iron core has a bigger hot spot size than the coil without an iron core, demonstrating the insignificance of the magnetic core at higher depths. In general, adding the ferromagnetic core results in a 1-2 mm^2^ reduction in the hot spot size in distances up to 5 mm from the head surface. The hot spot area at a depth of 2 mm, the cortical depth for rodents [44], is 3.6 mm^2^ showing the proposed coils’ suitability for rodent brain stimulation.

The supplementary video demonstrates the capability of the proposed coils (with a 5-degree tilting angle) to induce unilateral movements on an anesthetized rat with a voltage of less than 1 kV to reach the motor threshold [35] (available here). In this video, the twitch induced by focal stimulation occurs between the 2nd and 3rd second, showing the stimulation of the right-side hindlimb motor cortex, a small region in the brain in the 1 mm range [45]. Thus, the video shows that the proposed coils can reach the suprathreshold hindlimb motor cortex activation of small rodents.

## 3. Discussion

This work shows that adding ferromagnetic cores to the angle-tuned coils can reduce the field spread and accomplish up to 60% energy saving in close proximity of the coil (up to 5 mm) to reach the neural activation threshold in small diameter coils for rodent brain stimulation. The ferromagnetic cores can negatively impact depth performance and the inductance of the coils. However, for rodent brain stimulation, due to the small scalp-brain distance, the effect of depth requirement and inductance is not crucial. The proposed coils with/without a ferromagnetic core can easily reach a depth of 5 mm from the rodent’s head surface with desired focality and less unfavorable energy consumption for standard operation format like the theta-burst analysis. As a result, the proposed coils can be ideal tools for rodent brain stimulations.

## 4. Materials AND Methods

### 4.1. FEM Simulations

The FEM simulations were implemented using Comsol Multiphysics (COMSOL Multiphysics, Version 5.5). The head model is a homogenous and undifferentiated sphere with an electrical conductivity of 0.33 S/m. The spherical head model provides a reasonable comparison between the proposed and the existing coils without being biased toward a particular accurate head model.

The AC/DC module in COMSOL was used for all the simulations. The coil’s tilting angle varied from 0 to 60 degrees with 20-degree steps with different coil heights of up to 22 cm. The current excitation in all the coils is a sinusoidal wave with a frequency of 5 kHz. The calculation of the induced electric fields was performed in a three-dimensional space of a sphere with a diameter of 150 cm with the coil and the used head models located at the center of the sphere. To study the efficacy of the ferromagnetic core, an iron material with a relative permeability value of 1000 was used [14, 30], and its electrical conductivity was set to 0 to prevent the eddy current in the core. The diameter of the core varied for different coils to cover the gap inside the coil. Figure 2(a) illustrates the placement of the coil relative to the measurement cut-plane with the generated magnetic field, while Figure 2(b) shows the proposed coil design using the FEM simulations.

To compare the proposed coils’ performance with existing coils, we used a 17 cm diameter head model. A 5 mm thick insulation was considered to exist between the coil and the head model. We present the simulated data on the depth-spread tradeoff plot developed by Deng et al. [14], using the two best-fit curves for circular coils and figure-8 coils as the background. The proposed metrics in this plot rely on a normalized electric field to $E_{\max}$ [37] since the absolute value of the induced electric field strength can be tuned by the TMS stimulator output. One advantage of this depth-spread tradeoff representation is that it can guarantee the field strength between the coil and the head model. The cortical penetration depth surface is not higher than twice the field strength at the cortical penetration depth. This means that the risk of seizure accompanied by the stimulation is relatively low in the model introduced by Deng et al. [14].

### 4.2. Experimental Validation

We fabricated 8 prototypes using wire-wrapping around 3D printed coil holders [46]. The wires used for coil fabrication were made of Litz wire bundles with 135 pieces of insulated AWG30 wires. The prototypes had 2 different sizes and various tilting angles, and the electric field distributions were measured for each coil. The relative location of the coil to the measurement cut-plane is shown in Figure 2(a). The first set of coils has an inner and outer diameter of 1 cm and 3 cm, respectively, with 9 winding layers labeled “OD-3.” Note that the O.D. is an acronym for outer diameter, and the following number represents the coil’s outer diameter. The tilting angle varies from 0 to 60 degrees with a 20-degree step. The second coil, labeled as the “OD-9,” has an inner diameter and outer diameter of 3 cm and 9 cm, respectively, with 6 winding layers and a tilting angle of 40 degrees. Individual silicon steel sheets were stacked and used as ferromagnetic cores due to their high magnetic permeability and saturation values. The silicon steel sheets were insulated from each other to prevent the eddy current in the core. The core diameter for the OD-3 coil was 1 cm and for the OD-9 coil was 3 cm to fill the inner gap inside the coil. Figure 2(c) shows one of the fabricated prototypes.

A commercial Magstim 70-mm figure-8 coil (Magstim, Inc) was used for comparison. We also used a Magstim 200 (Magstim, Inc.) power supply to drive these coils at 30% of its maximum power output for all the measurements. The induced electric field distribution was measured with a spatial resolution of 5 mm with a high-spatial-resolution vector-field probe reported in our previous works [36]. The probe was first calibrated with an existing commercial coil at different driver power ratings. The purpose of the calibration and normalization is to obtain relative values to understand and verify the trends encountered in simulations, demonstrate the performance of the proposed coils in experimental setups, and identify the field distribution differences among the coils with different tilting angles. The absolute values for the electric field strength were measured at different depths for different coils and considered for validation purposes. As an example, the electric field strength value at a distance of 1.5 cm from the coil and a power rating of 30% of the Magstim 200 driver is 0.372 V for the 60-degree tilted coil with the ferromagnetic core and is 0.52 V for the commercial D-70 TMS coil. All the electric field measurements were performed in the air medium since the selection of the homogenous medium did not influence the electric field decay rate; this indicates that the electric field measurements in the air medium imitate the saline brain phantom. To map the vector field distribution and electric field decay rate, we recorded the amplitude of the electric field strength along the X, Y, and Z directions at each measurement point. The vector field strength was calculated by

(1)E=Ex2+Ey2+Ez2,

where Ex, Ey, and Ez are the measured electric field amplitude along the X, Y, and Z directions.

### 4.3. Analytical Comparisons

Simulations and experiments were performed with and without the ferromagnetic core to identify its effect on the induced electric field. The proposed coils’ depth-spread characteristics, including the electric field distribution, hot spot area, and the field intensity decay rate, were measured and analyzed and compared with conventional circular and figure-8 coils. The obtained data from simulations and experiments were normalized for easier comparison and independence from $E_{\max\text{’}\text{s}}$ absolute value, which can be regulated through the driver circuit.

Additional information regarding the COMSOL simulations details, and the experimental measurements procedures have been provided in the “Supplementary Data” (available here).

## Data Availability

The simulation and experimental data used to support the findings of this study are available from the corresponding author upon request.

## Supplementary Materials

Supplementary 1 Rat V-1 (Video): This supplementary video demonstrates the capability of the proposed coils (with a 5-degree tilting angle) to induce unilateral movements on an anesthetized rat with a voltage of less than 1 kV to reach the motor threshold.

Supplementary 2 Supplementary Data (Word File): This supplementary data provides additional information regarding the COMSOL simulations details and the experimental measurement procedures.
