# Supplementary material for: High-Performance Magnetic-core Coils for Targeted Rodent Brain Stimulations
Source: BME Front. 2022 Mar 5;2022:9854846. doi: 10.34133/2022/9854846 (PMC10521704; doi:10.34133/2022/9854846)
Supplement: Supplementary 2 — Supplementary Data (Word File): This supplementary data provides additional information regarding the COMSOL simulations details and the experimental measurement procedures. [file 9854846.f2.docx]

**SUPPLEMENTARY DATA**

1. **COLMSOL SIMULATIONS**

To perform the simulations, we used the AC/DC module in COMSOL. In all of the simulations and coil models, we assumed the same settings except the geometry of the coil, which was adjusted to improve the stimulation depth – focality characteristics. The calculation of fields was in the three-dimensional space of a sphere with a diameter of 150 cm with a mesh element size of ‘extra fine’ as defined by COMSOL. The coil and head models were located at the center of the sphere, and their sizes were much smaller than the diameter of the sphere to improve the accuracy of the calculations. As for the boundary conditions in the coil models, we used the default settings, which can be mathematically expressed as the followings according to the AC/DC module of the COMSOL user's guide [1]:

$$\boldsymbol{n}_{\boldsymbol{2}}\boldsymbol{\times}\left( \boldsymbol{E}_{\boldsymbol{1}}\boldsymbol{-}\boldsymbol{E}_{\boldsymbol{2}} \right)\boldsymbol{=0;}$$

$$\boldsymbol{n}_{\boldsymbol{2}}\boldsymbol{\cdot}\left( \boldsymbol{D}_{\boldsymbol{1}}\boldsymbol{-}\boldsymbol{D}_{\boldsymbol{2}} \right)\boldsymbol{=}\boldsymbol{\rho}_{\boldsymbol{s}}\boldsymbol{;}$$

$$\boldsymbol{n}_{\boldsymbol{2}}\boldsymbol{\times}\left( \boldsymbol{H}_{\boldsymbol{1}}\boldsymbol{-}\boldsymbol{H}_{\boldsymbol{2}} \right)\boldsymbol{=}\boldsymbol{J}_{\boldsymbol{s}}\boldsymbol{;}$$

$$\boldsymbol{n}_{\boldsymbol{2}}\boldsymbol{\cdot}\left( \boldsymbol{B}_{\boldsymbol{1}}\boldsymbol{-}\boldsymbol{B}_{\boldsymbol{2}} \right)\boldsymbol{=0,}$$

where ρ_s_ and **J_s_** denote surface charge density and surface current density, respectively, and **n_2_** is the outward normal from medium two.

All the COMSOL models' excitations were circular current excitations in sine waves with the same frequency of 5 kHz, corresponding to a pulse period of 200 μs. The consideration to use this frequency was based on the power spectrum analysis performed on the experimental stimulator (Magstim 200), which yielded a frequency of 5 kHz as shown in Figure 1 below. In addition, it has been previously shown that the monophasic pulse generated by Magstim 200 has a peak frequency component at nearly 5 kHz [2], with other works using a frequency of 5 kHz for their ferromagnetic core simulations [3–5].

To study the efficacy of the ferromagnetic core, an iron material with a relative permeability value of 1000 was used [3,6], and its electrical conductivity was set to 0 to prevent the eddy current in the core. Since we used a silicone steel core with a higher permeability in our experiments and we encountered linear coil performance with applied voltage with no saturation, we did not consider the possibility of saturation in our simulations. In addition, we used a lower permeability value in our simulations to demonstrate the performance of our coil in comparison to existing studies and to consider a worst-case scenario performance for our simulations.


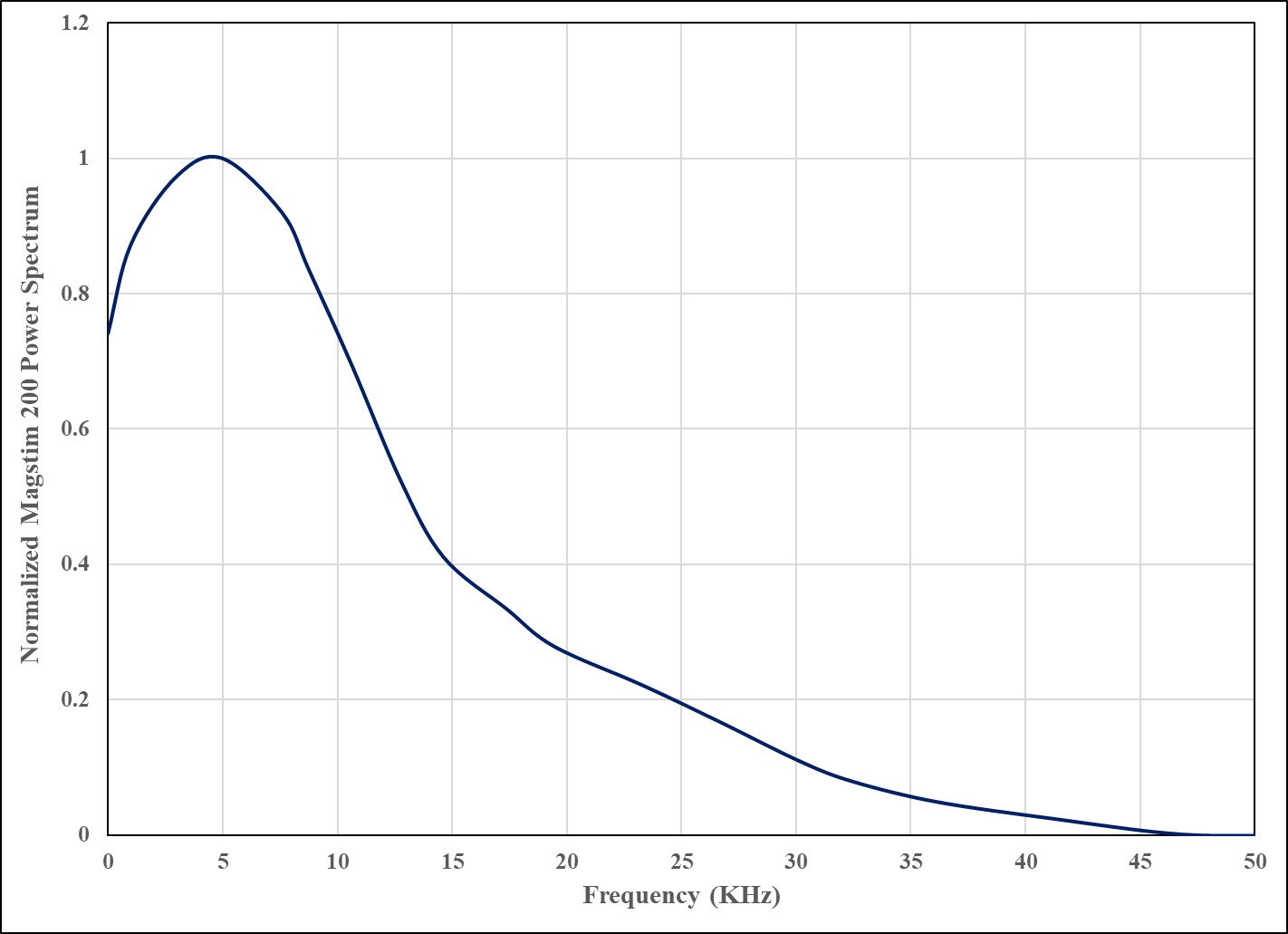


**Figure 1: The power spectrum for a 0.2 ms duration monophasic pulse of Magstim 200 obtained through Fast Fourier Transform (FFT).** The peak operation frequency of the Magstim 200 is shown to be close to 5 KHz.

1. **Experimental Measurements**

The coil was stabilized by a holder to eliminate the error in the measurements caused by the oscillations induced by the Lorentz force. The coil was directly loaded to a Magstim 200 stimulator. The stimulator's output power was set to 30% of its maximum output for all measurements, equivalent to about 500 V voltage on the coil. The electric field was measured in the air medium, considering that the field decays in a homogenous medium with a constant permittivity value, so the decay rate (percentage) doesn't change.

The ferromagnetic core used for the experimental measurement was silicon steel sheets due to their high magnetic permeability and saturation values. The silicon steel sheets were insulated from each other to prevent the eddy current in the core. The silicon steel sheets were 0.35mm (.014) M6 Grain Oriented Electrical Steel (GOES) from TC Metal. The material demonstrates a B_sat_ of 1.8 T and relative permeability of more than 5000.

A high-sensitivity electric field probe measured the induced electric field. The probe was fixed on a holder to avoid the vibration caused by the TMS pulses. The locations and movements of the probe referred to the coordinate underneath the probe holder. The signal detected by the probe displayed on the oscilloscope was the shape of the electric field pulse. The direction of the measured field was along the central axis of the probe's toroid ferromagnetic core. To map the vector field distribution, at each measurement point, we recorded the amplitude of the electric field along the X, Y (X and Y directions were within the plane that was perpendicular to the coils central axis) and Z directions. The electric field strength was then calculated by

$E= \sqrt{{Ex}^{2}+{Ey}^{2}+{Ez}^{2}}$,

Ex, Ey, and Ez are the measured electric field amplitude along the X, Y, and Z directions.

**REFERENCES**

[1] Multiphysics, C. O. M. S. O. L. AC/DC Module user’s guide. New York: COMSOL Multiphysics; 2013.

[2] Golestanirad L, Rouhani H, Elahi B, Shahim K, Chen R, Mosig JR, et al. Combined use of transcranial magnetic stimulation and metal electrode implants: a theoretical assessment of safety considerations. Phys Med Biol 2012;57:7813–27. https://doi.org/10.1088/0031-9155/57/23/7813.

[3] Deng Z-D, Lisanby SH, Peterchev AV. Electric field depth–focality tradeoff in transcranial magnetic stimulation: simulation comparison of 50 coil designs. Brain Stimulation 2013;6:1–13.

[4] Deng Z-D, Lisanby SH, Peterchev AV. Coil design considerations for deep transcranial magnetic stimulation. Clinical Neurophysiology 2014;125:1202–12.

[5] Gomez LJ, Goetz SM, Peterchev AV. Design of transcranial magnetic stimulation coils with optimal trade-off between depth, focality, and energy. Journal of Neural Engineering 2018;15:046033.

[6] Salvador R, Miranda PC, Roth Y, Zangen A. High-permeability core coils for transcranial magnetic stimulation of deep brain regions. 2007 29th Annual International Conference of the IEEE Engineering in Medicine and Biology Society, IEEE; 2007, p. 6652–5.
